# Soy-Based Tempeh Rich in Paraprobiotics Properties as Functional Sports Food: More Than a Protein Source

**DOI:** 10.3390/nu15112599

**Published:** 2023-06-01

**Authors:** Dionysius Subali, Revelo Eved Christos, Vasya Theodora Givianty, Alberta Valencia Ranti, Felicia Kartawidjajaputra, Lina Antono, Rendy Dijaya, Nurpudji Astuti Taslim, Gianluca Rizzo, Fahrul Nurkolis

**Affiliations:** 1 Department of Biotechnology, Faculty of Biotechnology, Atma Jaya Catholic University of Indonesia, Jakarta 12930, Indonesia; dionysius.subali@atmajaya.ac.id (D.S.); reveloevedchristos@gmail.com (R.E.C.); vasyatheodora@gmail.com (V.T.G.); valenciaranti@gmail.com (A.V.R.); 2 Health and Nutrition Science Department, Nutrifood Research Center, PT Nutrifood Indonesia, Jakarta 12930, Indonesia; felicia@nutrifood.co.id (F.K.); lina.antono@nutrifood.co.id (L.A.); rendy.dijaya@nutrifood.co.id (R.D.); 3 Division of Clinical Nutrition, Department of Nutrition, Faculty of Medicine, Hasanuddin University, Makassar 90245, Indonesia; pudji_taslim@yahoo.com; 4 Independent Researcher, Via Venezuela 66, 98121 Messina, Italy; 5 Department of Biological Sciences, Faculty of Sciences and Technology, State Islamic University of Sunan Kalijaga, Yogyakarta 55281, Indonesia

**Keywords:** tempeh, antioxidants, soybean, sports food, fermented soy, functional food, paraprobiotics

## Abstract

To date, there has been no recent opinion that explores tempeh as a functional food that can improve sports performance. Hence, this opinion article aims to elaborate on recent findings on the potential effect on sports performance of soy-based tempeh. This opinion paper presents updated evidence based on literature reviews about soy-based tempeh and its relationship with sports performance. The paraprobiotic role of *Lactobacillus gasseri* for athletes has been found to restore fatigue and reduce anxiety. This is achieved by increasing protein synthesis activity in eukaryotic initiation factor-2 (EIF2) signaling known as an adaptive pathway for integrated stress response. In addition, these paraprobiotics prevent down-regulation associated with the oxidative phosphorylation gene, thereby contributing to the maintenance of mitochondrial function and recovery from fatigue. The authors believe that this opinion article will encourage researchers to continue to evolve soybean-based tempeh food products and increase the performance of athletes by consuming soy-based foods.

## 1. Introduction

Muscle recovery and muscle performance are important for athletes, in both endurance or resistance training [1]. To optimize these aspects, nutritional needs relating to macronutrients and micronutrients must be fulfilled. Athletes’ requirements for carbohydrates, protein, and fat are different from those of people in general and range from 8.0 to 10.0 g/kg, 1.5 to 2.0 g/kg, and 0.5 to 1.0 g/kg, respectively [2,3].

Protein is one of the most consumed nutrients by athletes to encourage muscle growth and repair [4,5]. Furthermore, many athletes consume a high amount of protein, and this high-protein diet is also commonly associated with muscle strength and hypertrophy [5]. In addition, a high-protein diet is now recommended for recovery from intense sports or injuries [5,6]. Dietary protein promotes the remodeling of muscle and body proteins by providing essential amino acids [4]. Protein is needed in large quantities to maintain the body’s condition when participating in sports requiring extreme metabolic stress. Moreover, competitive stress requires not only a great deal of energy but also large quantities of protein to manage the body’s needs. Protein also plays an important role in the metabolism of hemoglobin, which strongly correlates to performance [7]. This is because hemoglobin is a protein that functions to transport oxygen molecules to various parts of the body through blood circulation, and it plays a crucial role in cardiovascular fitness or maximum oxygen volume (VO_2_Max) levels [8]. An increase in VO_2_Max is associated with high levels of hemoglobin [9].

Currently, sports food is still dominated by animal-based ingredients, such as meat, milk, and eggs, but the use of animals as raw material for sports nutrition causes problems. For example, the use of plants as animal feed is wasteful. Replacing animal-based items with plant-based alternatives would increase food availability equivalent to the dietary needs of approximately 350 million people by reallocating feed for human consumption [10]. Moreover, the use of meat causes greenhouse gas emissions (GHGEs). Although the large scale of the issue makes precise figures difficult to determine, it is estimated that the percentage of emissions from poultry is ~10% while emissions from cattle contribute ~60% (red meat emits six times more GHGEs than poultry) [11]. In addition, there are side effects that can affect the health of athletes. Some athletes have a deficiency in digesting lactose in milk due to a genetically determined progressive decline in lactase expression after weaning, known as lactase non-persistence [12]. High consumption of red meat also affects athletes’ health, because it contains high saturated fat and is linked to causing colon and rectal cancer [13]. Therefore, sports food from natural ingredients, such as plant-based food, which is abundant and less expensive, needs to be developed. Notably, soybean is the one of main sources of vegetable protein for humans, which is also part of the grains. According to a literature review conducted by Guo et al. in 2020, soybeans from traditional farming have a significant protein content of approximately 36–38% [14]. After being fermented, the protein content in soybeans does not change significantly and remains at around 44% [15]. Significant changes only occur in the carbohydrate content because of the enzymes secreted by *R. microsporus* var. *oligosporus*.

In the world of sports, a fast recovery process is also crucial to improve sports performance by avoiding muscle tissue damage [16]. As explained above, vegetable protein is no less effective than protein obtained from meat [14,17]. Such vegetable protein can be obtained from tempeh, a traditional food commonly consumed in Indonesia [18,19]. In a study, it was shown that resistance exercise, especially eccentric exercise, causes muscle damage, as evidenced by increasing creatine kinase (CK) levels, muscle soreness, and reduced muscle strength [20]. The study compared the effectiveness of post-exercise protein intake between whey, placebo, and a tempeh drink. The results show that the consumption of a tempeh drink post-exercise lowered CK levels more than the placebo group. Twenty-four hours after exercise, it was also found to improve muscle strength and muscle soreness. Moreover, tempeh is characterized by high antioxidant activity, which is important for reducing oxidative stress and enhancing performance. Tempeh, therefore, has significant potential for use in a sports drink to reduce CK levels, improve muscle strength, and reduce muscle soreness after resistance exercise [20].

Tempeh is made by fermentation using beneficial microorganisms such as mold and lactic acid bacteria (LAB). LAB has potential as a probiotic. However, generally, people consume cooked tempeh. Thus, the microorganisms are inactivated and become paraprobiotics. The paraprobiotics in tempeh can induce immune gene expression in, for example, the IgA gene, which therefore produces more IgA, an antibody that acts as a defense against the presence of antigens in the mucosal membrane [21]. Thus far, review articles have discussed tempeh only in terms of health, and no recent study has explored tempeh as a functional food that can improve sports performance. Hence, the aim of this article is to elaborate on recent findings regarding the potential effect on sports performance of soy-based tempeh. This paper presents updated evidence based on literature reviews regarding soy-based tempeh and its relationship with sports performance. The authors believe that with this article, researchers can continue to develop soybean-based tempeh food products and thereby contribute to enhancing the performance of athletes.

## 2. Soy-Based Tempeh in General

**Figure 1:**
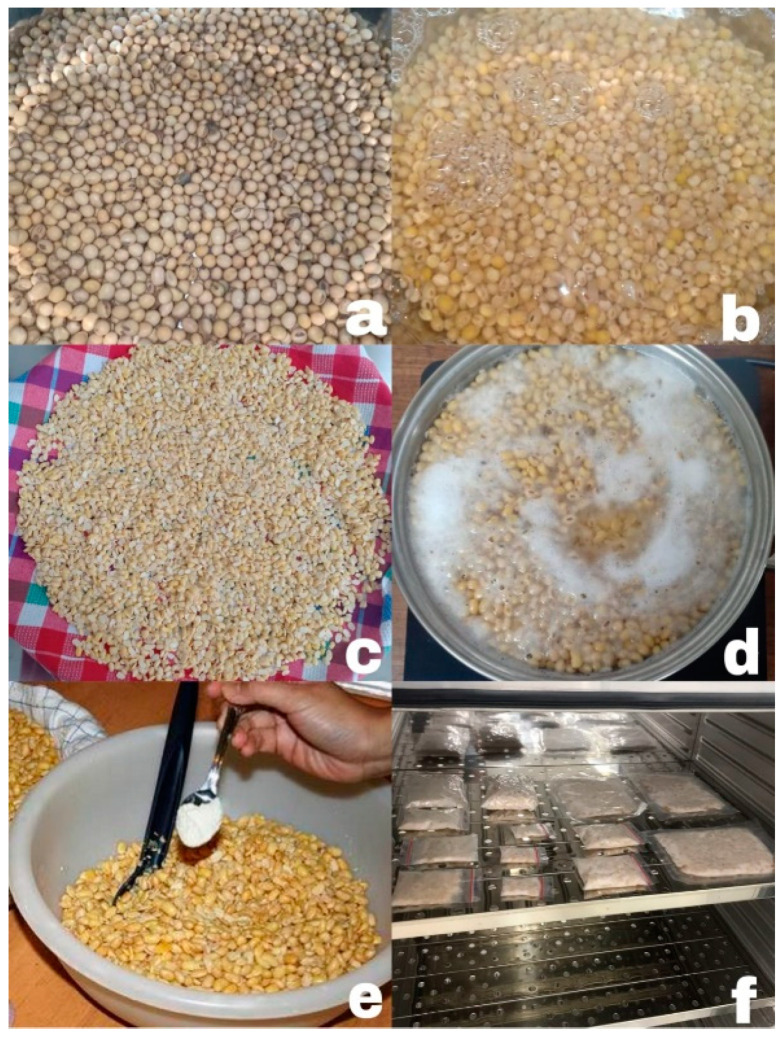
Tempeh production methods (**a**) washing, (**b**) soaking in water, (**c**) dehulling, (**d**) boiling, (**e**) inoculating with starter, (**f**) packaging, and incubating.

The use of soybeans (*Glycine max* (L.) Merr.) for making tempeh is well known. However, other legumes, such as corn, red beans, green beans, and black beans can also be used as a substrate for tempeh. Figure 1 shows that the standard process of making tempeh involves washing, soaking in water, dehulling, boiling, inoculating with starter, packaging, and incubating [22]. *Rhizopus microsporus* var. *oligosporus,* which is the main mold used in tempeh, secretes several enzymes such as α-amylase, cellulase, esterase, β-glycosidase, and protease, which break down the macromolecules in soybean into small molecule nutrients and several secondary metabolites with functional properties [23]. *R. microsporus* var. o*ligosporus* was found abundantly on soil or *Hibiscus* leaves [24]. 

Lactic acid bacteria have also been found in tempeh and contribute to the tempeh-making process and tempeh nutrition and flavor [25,26]. In one study, all the ingredients, from raw soybean and freshwater that have been used to soak soybeans, starters, and tempeh were investigated. The researchers found lactic acid bacteria during the fermentation process. Some of these bacteria contribute to the nutritional content of tempeh (Table 1). For example, *Klebsiella pneumoniae* produces B_12_. They also found *Lactobacillus* in the tempeh with double boiling treatment [25]. This conclusively demonstrates the presence of paraprobiotics in tempeh. 

**Figure 2:**
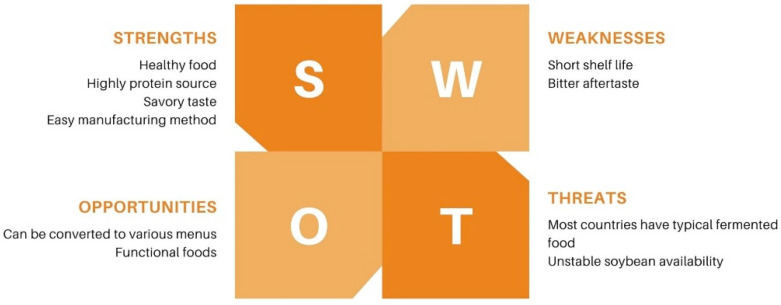
SWOT analysis of commercial tempeh.

Tempeh is very popular worldwide due to its beneficial effects on health, its taste, its convenience, especially for vegetarians, and its affordability [22]. In addition, tempeh industries have been established worldwide such as in Spain, Japan, Italy, North America, the Netherlands, and Australia [28]. Figure 2 shows that tempeh has a high commercial value. In addition, making tempeh is relatively easy. Tempeh can also be made into various menu variants and functional foods. Moreover, various studies have shown that tempeh is a strong candidate for sports food due to its high protein, vitamin, antioxidant, probiotic, and calcium contents [15].

## 3. Tempeh as Nutrient Source for Athletes

**Figure 3:**
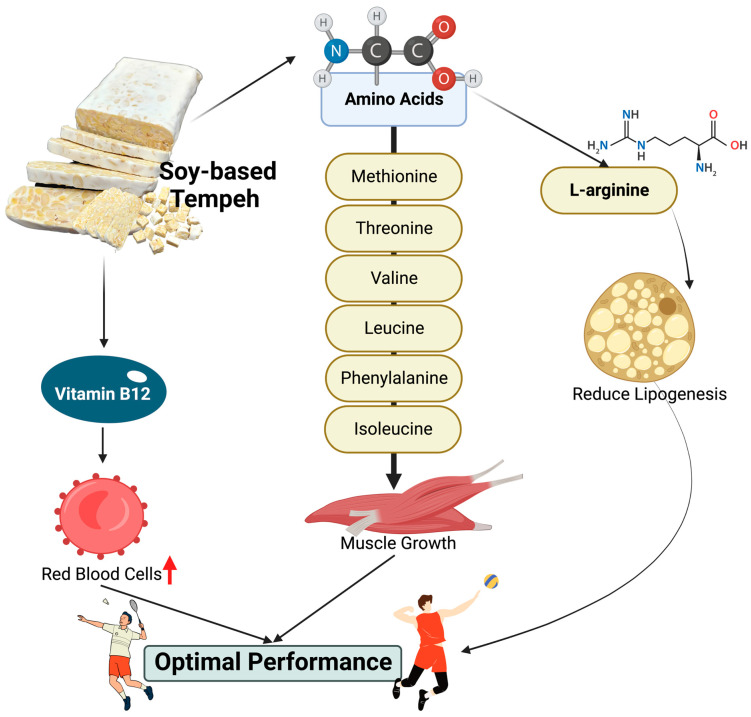
Amino acids and vitamin B12: the mechanism of tempeh in performance optimization. Created with BioRender.com premium license by Fahrul Nurkolis.

Athletes have been searching for the best food candidate for many years to enhance their sports performance and recovery. One of the nutrients that athletes need is protein, either for resistance or endurance training. Specifically, athletes need 1.5–2.0 g of protein/kg body weight daily to induce muscle synthesis and recovery [2]. Some amino acids contained in tempeh have been found to help muscle growth, such as methionine, threonine, valine, leucine, phenylalanine, and isoleucine (Figure 3) [29,30]. Interestingly, one of the abundant amino acids in tempeh is L-arginine [31,32]. L-arginine is beneficial in improving fat levels in the blood by reducing fat formation in the body (lipogenesis) (Figure 3) [33]. More interestingly, a recent systematic review and meta-analysis study showed that L-arginine supplementation can enhance anaerobic performance in athletes [33].

Vitamin B12 deficiency is a common problem worldwide, in countries such as India, Mexico, Central and South America, and areas in Africa, and it is also common among vegetarians in Asia [34]. One cause of this is the limited number of dietary sources of vitamin B12, especially in plant-based food. Vitamin B12 deficiency leads to a number of diseases, such as pernicious anemia, megaloblastic anemia, and hyperhomocysteinemia, and it can be fatal [35]. Athletes and coaches are typically aware that for optimal performance, an increase in red blood cells is needed (Figure 3). Therefore, vitamin B12 is a supplement that is commonly used in sports. However, even though vitamin B12 is quite popular in sports, there is no study on the correlation between vitamin B12 and red blood cells [36]. Tempeh is one of the plant-based fermented foods that contain vitamin B12. Vitamin B12 is produced by the contamination of *Klebsiella pneumoniae, Citrobacter freundii*, or *Propionibacterium*. These microorganisms can be added as a co-culture or can be found in freshwater during the soybean-soaking process. The *K. pneumoniae* in tempeh does not produce enterotoxin since the virulence-associated gene, *rmpA*, is absent. Thus, vitamin B12 is not pathogenic [27,35]. Approximately 60% of soybeans in Indonesia are used to make tempeh. As tempeh is a result of fermentation, nutrients and bioactive components that are beneficial for the body can be easily absorbed [37]. Based on these data, tempeh is one of the most promising vegan protein sources for athletes.

## 4. The Paraprobiotic Properties of Tempeh for Athletes

**Figure 4:**
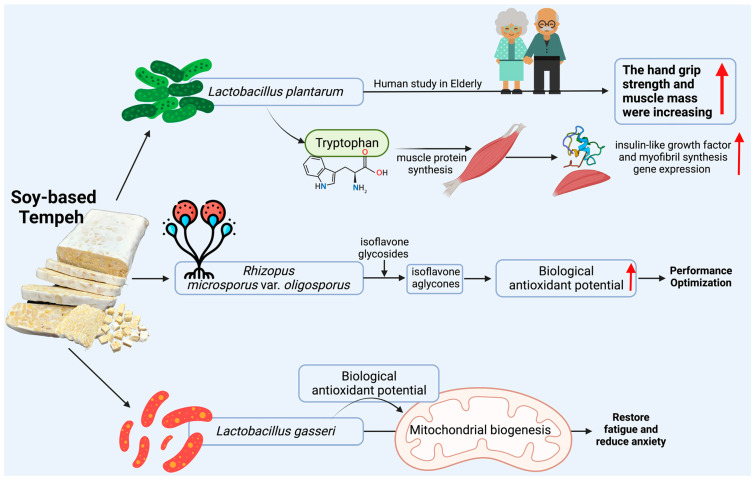
Functional paraprobiotics found in tempeh and their beneficial biomechanism for athletes. Created with BioRender.com premium license by Fahrul Nurkolis.

For more than ten years, the potential health benefits of probiotics have been studied. Probiotics can be obtained from tempeh, and have various benefits for the gastrointestinal microbiota ecosystem, stimulate the immunological system and anticarcinogenic activity, and reduce oxidative stress. *Lactobacillus* and *Bifidobacterium* are the most studied probiotics. In humans, the presence of *Lactobacillus* species is normal and non-pathogenic for the maintenance of the intestinal microbial ecosystem. *Lactobacilli* are also known to produce several antimicrobial compounds and have inhibitory activity against enteropathogenic multiplication. The effect of a probiotic, *Lactobacillus plantarum,* on improving muscle mass has been studied. In the study, *L. plantarum* was given as a supplement to older adults. The results show that after probiotic supplement consumption for 18 weeks, hand grip strength and muscle mass increased (Figure 4). This probiotic supplement can also improve gut microbiota and influence muscle protein synthesis and decomposition by means of, for example, tryptophan. Tryptophan is known as a substrate for muscle protein synthesis, which can stimulate the insulin-like growth factor pathway in muscles and increase the myofibril synthesis gene expression (Figure 4) [38]. The study also found that *L. plantarum* benefits athletes in terms of muscle mass.

Athletes are often exposed to excess circulating reactive oxygen species (ROS) due to intense physical activity and increased oxygen consumption, which results in the necrosis and degeneration of liver and kidney cells. Isoflavones are antioxidant compounds found in soybeans that can counteract free radicals through their active mechanisms [39]. After being fermented into tempeh, *R. microsporus* var. *oligosporus* secretes β-glycosidase, which converts isoflavone glycosides into isoflavone aglycones. Isoflavone aglycones have higher antioxidant activities than isoflavone glycosides (Figure 4) [40]. Several studies show that some probiotic strains reduce systemic oxidative stress. One study found that the level of reactive oxygen metabolites (ROMs) in the control group (not given probiotics) was significantly higher than in the probiotic group after exercise [39]. Moreover, the biological antioxidant potential (BAP) level was significantly increased after probiotic supplementation. BAP levels were also significantly higher in the probiotic group than in the control group after exercise. 

Tempeh consumption is mostly in a cooked condition, and as a result, the probiotics in tempeh become paraprobiotics, which are inactivated microbial cells (non-viable). Paraprobiotics can be used as a solution to replace the use of probiotics that have drawbacks, such as industrial processing, and problems with storage, by enabling cell viability to be maintained. They can also function to limit the risk of probiotic consumption by vulnerable people, such as the elderly or individuals with immune deficiencies [41]. Paraprobiotics can help to regulate the adaptive and innate immune systems, have antioxidant, anti-inflammatory, and antiproliferative properties, and exert antagonistic effects against pathogens [42,43]. The effect of paraprobiotics, *L. fermentum,* on the production of neuromodulators by inhibiting acetylcholinesterase (AChE) activity has been studied. However, the specific mechanism is not known [44]. However, developments regarding paraprobiotics to improve sports performance need further research because they are still very limited. As paraprobiotics, dead probiotics in tempeh will lyse, causing the breakdown of peptidoglycan and lipopolysaccharide (LPS). These components act on pattern recognition receptors (PRRs) consisting of TLRs and nucleotide-binding domain (NOD) proteins. These receptors are the main sensors for the innate immune system. TLR4 is the main receptor for recognizing LPS, while NOD1 and NOD2 recognize muramyl peptides produced by peptidoglycan [45]. 

For athletes, an increase in fatigue is a serious problem, in addition to a decrease in immunity. The close relationship between immunity and fatigue may be the cause of chronically fatigued patients’ susceptibility to viral infections [46], especially since high-intensity exercise (HIE) can increase the risk of upper respiratory tract infections (URTIs). Some studies have shown that the concentration of salivary secretory immunoglobulin A (sIgA) and natural killer cells, commonly immunocytes against viral and bacterial infections, decreased after prolonged HIE [47]. Dendritic cells (DCs) have essential roles in the immune system, such as antigen presentation. DCs are divided into two groups, pDC (plasmacytoid DC) and mDC (myeloid DC), based on phenotype and function. pDC is related to antiviral response, whereas mDC is related to bacterial infection response. Komano et al. conducted a study on the efficacy of heat-killed *Lactococcus lactis* on immunity and fatigue during consecutive high-intensity exercise in male athletes [4]. *Lactococcus lactis* JCM 5805 (LC-Plasma), a unique LAB that can activate plasmacytoid dendritic cells (pDC), was used. The study aimed to evaluate the effect of LC-Plasma on dendritic cells (DC), subjective indices of upper respiratory tract infection (URTI) and fatigue in athletes with HIE. The researchers compared placebo-controlled and LC-Plasma capsules containing 100 billion cells of heat-killed LC-Plasma. The article hypothesizes that the ingestion of LC-Plasma could maintain pDC activity and repress infection morbidity even during consecutive HIE. Furthermore, the researchers also hypothesized that LC-Plasma intake is effective for overcoming fatigue accumulation in athletes. The result showed that LC-Plasma supplementation increased the pDC maturation marker (CD86) compared to the placebo group for 13 days, and URTI symptoms decreased. Moreover, LC-Plasma ingestion was found to decrease cumulative days of fatigue. The fatigue accumulation in the LC-Plasma group was found to be lower than in the placebo group. From these results, it is evident that the supplementation of LC-Plasma can prevent URTI infection through pDC activation and decrease the accumulation of fatigue.

The paraprobiotic role of *Lactobacillus gasseri* for athletes has been found to restore fatigue and reduce anxiety (Figure 4) [48]. *Lactobacillus gasseri* functions to increase protein synthesis activity in EIF2 signaling known as an adaptive pathway for integrated stress response. In addition, these paraprobiotics prevent down-regulation associated with the oxidative phosphorylation gene, thereby contributing to the maintenance of mitochondrial function and recovery from fatigue [49]. Another study found that heat-killed *Bifidobacterium breve* caused an increase in the induction of mitochondrial biogenesis in muscles through AMPK-PGC-1α signaling, thereby increasing muscle mass and providing anti-obesity effects [50].

## 5. Discussion, Future Direction, and Implication

**Figure 5:**
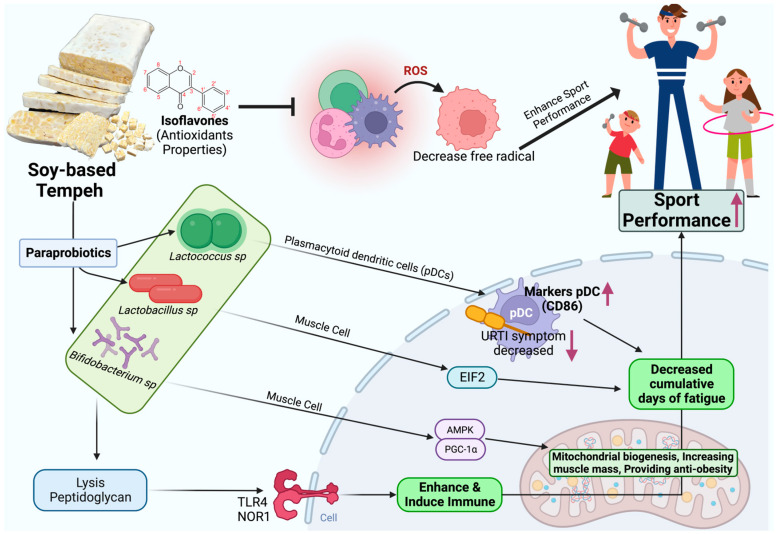
Predicted mechanism of sport-enhancing properties from soy-based tempeh and their future meal product innovation. ROS: reactive oxygen species; EIF2: eukaryotic initiation factor-2, AMPK: 5’ AMP-activated protein kinase, PGC-1α: peroxisome proliferator-activated receptor-γ coactivator-alpha, TLR4: Toll-like receptor 4, NOR1: oxidored-nitro domain-containing protein 1, pDC: plasmacytoid dendritic cell, URTI: upper respiratory infection. Created with BioRender.com premium license by Fahrul Nurkolis.

Tempeh has many ingredients that are beneficial for athletes (Figure 5). Mold and LAB, which are both found in tempeh, are known probiotics. Tempeh is usually consumed after first being cooked. However, heat causes the probiotics to become inactive (paraprobiotics). Although paraprobiotics are inactivated bacteria, paraprobiotics in tempeh have many health benefits. A number of studies have established that paraprobiotics have an important role in the gastrointestinal microbiota ecosystem, the stimulation of the immunological system, reducing oxidative stress, reducing fatigue, and increasing muscle mass in different pathways. The paraprobiotics contained in tempeh can help athletes improve their sports performance via the activation of pDC. Heat-killed LC-Plasma supplementation reduces URTI morbidity and symptoms during consecutive HIE. Further benefits of heat-killed LC-Plasma include immune reduction and decreased fatigue. Ripe tempeh produces paraprobiotics that can help increase muscle mass and reduce fatigue (Figure 5). *L. gasseri* and *B. breve* have been shown to assist biogenesis and enhance mitochondrial function through EIF2 and AMPK-PGC-1α signaling. This review article promotes the consumption of tempeh as a functional food to enhance sports performance. The peptidoglycan and lipopolysaccharide produced by paraprobiotics also play a role in TLR and NOR receptors which are the main sensors of the innate immune system. A study also showed that daily consumption of 100 g of steamed tempeh for 16 days enhanced IgA production and enhanced the *Akkermansia muciniphila* number, which can act as improvement markers for lower type 2 diabetes and obesity [51]. The increase in *A. muciniphila* is caused by polyphenol compounds contained in tempeh. An increase in IgA, particularly sIgA (secretory IgA), from eating tempeh also can decrease the risk of being infected by URTI (upper respiratory tract infection), which often occurs among athletes or individuals who frequently exercise in a vigorous or high intensity [52]. 

As a traditional food, tempeh is very popular and accessible. It is also a diverse food, which can be served as a snack bar made from tempeh flour. Tempeh snack bars consisting of 20% tempeh flour and 42% cornstarch and other additives are rich in proteins and carbohydrates [53]. Such snack bars can be an alternative to other healthy snacks and are quick to consume. Another tempeh-based product is tempeh flour. A previous study highlighted that the combination of tempeh with eel as a functional flour produces beneficial effects on nutritional status biomarkers [54]. In addition to being a food ingredient, tempeh can be consumed in the form of drinks, such as tempeh milk, which is made with tempeh extract and mixed with a variety of flavors, such as chocolate, vanilla, and strawberry. This tempeh milk powder is a non-fat milk powder and has a nutritional content that is not inferior to ordinary tempeh [55]. Moreover, it can be consumed anywhere and at any time in athletes’ busy schedules. 

This article is expected to encourage further research on tempeh, because there are still many potential aspects that can be explored, especially in the fields of microbiomes and transcriptomics. Specifically, a proteomic study that explores muscle synthesis and mitochondria biogenesis activities of the paraprobiotics from tempeh as a sports food in detail is needed.

## Figures and Tables

**Table 1:** Microorganisms produced during tempeh fermentation.

Phylum	Species	Role	Sources
Firmicutes	*Clostridium beijerinckii*	Contaminant from raw soybean; inactivated during the tempeh production process	[27]
Firmicutes	*Lactococus taiwanensis*		
Proteobacteria	*Acetobacter indonesiensis*	Produce acetic acid to prevent spoilage and pathogen bacteria growth	[26,27]
Proteobacteria	*Acetobacter aceti*		
Firmicutes	*Lactobacillus fermentum*	Produce lactic acid to prevent spoilage and pathogen bacteria growth; paraprobiotic	
Firmicutes	*Lactobacillus delbrueckii*		
Firmicutes	*Lactobacillus mucosae*		

## Data Availability

Not applicable.
